# O046: Impact of infections due to carbapenem resistant gram negatives on length of hospitalisation: multi state model approach

**DOI:** 10.1186/2047-2994-2-S1-O46

**Published:** 2013-06-20

**Authors:** G De Angelis, B Fiori, T Spanu, E Tacconelli

**Affiliations:** 1 Catholic University, Rome, Italy; 2 Universitätsklinikum Tübingen, Tübingen, Germany

## Introduction

Infections caused by gram negative bacteria resistant to carbapenems (CRGNinf) represent a growing problem in many countries, with mortality rates reaching 50% in several reports. The economic impact in term of associated extra length of hospital stay (LoS) is less documented, with most of evidence relying on case-controls studies in intensive care unit.

## Objectives

Multistate modelling has been reported to offer a precise estimation of LoS associated to hospital acquired infections, limiting the effect of bias of observational studies.

## Methods

Data of 58,646 patients hospitalised in 2011 at a 1200-bed tertiary care hospital in central Italy were retrospectively collected. A multistate model in which the occurrence of CRGNinf was the time-dependent exposure and discharge or death was the study endpoint was designed. The excess LoS associated to CRGNinf was extracted computing the Aalen-Johansen estimator of the matrix of transition probabilities. Multivariate Cox regression analysis was used to assess the independent effect of CRGNinf on excess LoS. Variables for adjustment included demographic data, co-morbidities and concurrent bloodstream infections.

## Results

Ninety-seven CRGNinf were detected (0.2%), 40 (41.2%) associated with patient’s death. CRGNinf prolonged LoS by 14.2 days (95% confidence interval (CI) 8.1-20.2) compared to uninfected patients. The inclusion of CRGNinf as a time-dependent variable in a multivariate Cox proportional hazards model confirmed that the occurrence of infection significantly reduced the hazard of end of hospital stay and consequently prolonged LoS in hospital compared to uninfected patients (hazard ratio (HR) 0.60, 95% CI 0.49-0.74). When stratifying the analysis by outcome (discharge or death), CRGNinf was associated with a further reduced risk of discharge from hospital (adjusted HR 0.39, 95% CI 0.30-0.51) and, in parallel, to a significant higher risk of end of hospital stay because of death (adjusted HR 3.45, 95% CI 2.49-4.77).

## Conclusion

This is the first multi-state model study to estimate the excess LoS associated with CRGNinf in a large hospital cohort. The high mortality of patients suffering this infection does not impact on LoS, which is confirmed to be significantly longer compared to CRGN uninfected patients.

## Disclosure of interest

None declared.

